# Do circulating neutrophil extracellular traps predict recurrence in early breast cancer?

**DOI:** 10.3389/fonc.2022.1044611

**Published:** 2023-01-16

**Authors:** Bertha Alejandra Martinez-Cannon, Karen Garcia-Ronquillo, Monica M. Rivera-Franco, Eucario Leon-Rodriguez

**Affiliations:** ^1^ Hematology-Oncology Department, Instituto Nacional de Ciencias Medicas y Nutricion Salvador Zubiran, Mexico City, Mexico; ^2^ Eurocord, Hôpital Saint-Louis APHP, Institut de Recherche de Saint-Louis (IRSL) EA3518, Université de Paris Cité, Paris, France

**Keywords:** neutrophil extracellular traps, NETs, NETosis, breast cancer, recurrence

## Abstract

**Background:**

Neutrophil extracellular traps (NETs), three-dimensional structures formed by neutrophil enzymes such as neutrophil elastase (NE) and nuclear components (DNA), have been associated with progression and metastasis in breast cancer (BC). Thus, the aim of this study was to evaluate the association of circulating NETs with clinicopathological characteristics and outcomes in early BC.

**Methods:**

A prospective cohort included women with newly diagnosed early BC. NETs were defined as the presence of NE-DNA complexes in plasma, measured by optical density. Levels of NETs were dichotomized according to the median, as low and high levels of circulating NETs. Fisher’s exact test was used to evaluate associations between NETs and clinicopathological characteristics and outcomes. Survival was assessed using the Kaplan Meier method and log-rank test.

**Results:**

Forty patients were included, 23 (57.5%) patients with low and 17 (42.5%) with high levels of circulating NETs. No associations were found between clinicopathological characteristics and circulating NETs levels. Recurrence (p = 0.99) and site of recurrence (p = 0.99) were not statistically associated with plasma NETs levels. Overall, recurrence-free survival was not statistically different between circulating levels of NETs.

**Conclusions:**

With a short follow-up and low number of events, our results suggest that circulating levels of NETs at diagnosis of early BC are not associated with more aggressive clinicopathological characteristics, recurrence, or site of recurrence.

## Introduction

Globally, breast cancer (BC) is the most frequently diagnosed malignancy and the leading cause of cancer-related death in women (1). Although current treatments have improved survival in patients with early BC, tumor recurrence and metastases are the leading cause of mortality in patients with BC (2). Currently, many prognostic biomarkers for recurrence of BC have been validated, especially for hormone receptor (HR) positive (HR+) BC. Genomic signatures such as the Oncotype Dx recurrence score (RS) (Genomic Health), PAM50-based Prosigna risk of recurrence (ROR) (NanoString), Breast Cancer Index (BCI) (bioTheranostics), EndoPredict (EPclin) (Myriad Genetics), and MammaPrint 70-gene signature (Agendia BV) have been proven helpful in identifying patients with HR+ BC who are at increased risk of recurrence and may benefit from adjuvant chemotherapy (3). Although traditional biomarkers such as tumor size, nodal status, tumor grade, HR expression, and HER2 expression may provide predictive and prognostic information, our ability to predict recurrence in early BC is far from accurate. Thus, identifying biomarkers that could predict outcomes is of critical importance.

A growing number of prognostic and predictive biomarkers are currently being investigated (4). Neutrophil extracellular traps (NETs) are three-dimensional structures formed by decondensed chromatin, histone, deoxyribonucleic acid (DNA), and neutrophil granular proteins such as neutrophil elastase (NE), a serine protease, and myeloperoxidase (MPO). The role of NETs in cancer progression and metastasis is currently being studied in multiple tumor types (5). Prior studies in murine and *in-vitro* models have demonstrated that NETs are associated with progression of disease in lung, colorectal, and other cancer types (6). In BC, high NETs levels have been associated with disease progression, metastasis, and vascular complications such as venous thromboembolism (7). Furthermore, in a previous study by our group, higher levels of circulating NETs were observed in patients with locally advanced and metastatic disease as compared to women with localized BC (8). Therefore, the main objective of the present report was to evaluate the association of circulating NETs with clinicopathological characteristics and recurrence specifically in patients with early BC.

## Methods

A prospective cohort included 45 women with newly diagnosed BC at the *Instituto Nacional de Ciencias Medicas y Nutricion Salvador Zubiran* (INCMNSZ) from May 5^th^, 2017, to January 1^st^, 2019. Patients with relapsed disease, previous treatment, auto-immune disease, thrombotic disease, active infection, and previous history of cancer were excluded. Study procedures were approved by the Institutional Review Board at the INCMNSZ and written consent was obtained from all participants prior to study inclusion. Results regarding patients with early, locally advanced, and metastatic BC from this prospective cohort have been previously reported. (8) For this analysis only patients with early BC (stages I – III) from the previously reported cohort were included (8).

Patients’ records were reviewed to obtain sociodemographic, clinical and pathological characteristics, and survival outcomes including recurrence and death. Detailed procedures regarding the detection and quantification of NETs have been previously described (8). Briefly, peripheral blood was collected at diagnosis prior to any treatment initiation. Using enzyme-linked immunosorbent assay (ELISA), plasma levels of NE-DNA complexes were analyzed using a polyclonal antibody to NE (Cloud-Clone, PAA181Hu01) and a peroxidase labeled anti-DNA monoclonal antibody (component no.2 of the commercial Cell Death Detection ELISA PLUS, Roche). NETs were defined as the presence of NE-DNA complexes in plasma, measured by optical density at 450 mm wavelength.

Levels of NETs were dichotomized according to the median (0.6705) of the entire cohort (8). Consequently, patients were classified as having low (<0.6705) and high levels (>0.6705) of circulating NETs. Descriptive statistics were used to analyze clinicopathological characteristics and outcomes. Fisher’s exact test was used to evaluate associations between circulating levels of NETs and clinicopathological characteristics and outcomes. Recurrence-free survival (RFS) was assessed using the Kaplan Meier method and log-rank test. A value of *p <*0.05 was considered statistically significant. Statistical analyses were performed using SPSS (Statistical Package for the Social Sciences) v25.

## Results

Forty patients were diagnosed with early-stage BC and were included in the current analysis. The median age was 56.5 years and most of the patients (52.5%) had stage II disease. The most frequent histology was ductal carcinoma (87.5%). The majority (72.5%) expressed HR, 12.5% expressed HER2, and 17.5% were triple negative. The majority had grade 2/3 (85%) tumors and more than half (55%) had a Ki-67 of less than 30%. Twenty-three (57.5%) patients were classified as having low levels of circulating NETs and 17 (42.5%) as having high levels of circulating NETs. **Table 1** lists patients’ characteristics in the overall population.

**Table 1:** Patients’ characteristics.

	Overall population (n = 40)
Age in years (median)	56.5
Stage	
Early (I – IIA)	27 (67.5%)
Locally advanced (IIB – IIIC)	13 (32.5%)
Histologic subtype	
Ductal	35 (87.5%)
Lobular	5 (12.5%)
Hormone receptor (HR) expression	
HR positive (HR+)	29 (72.5%)
HR negative (HR-)	11 (27.5%)
HER2 expression	
HER2 positive (HER2+)	5 (12.5%)
HER2 negative (HER2-)	35 (87.5%)
Molecular subtype	
HR+/HER2-	28 (70%)
HR+/HER2+	1 (2.5)
HR-/HER2+	4 (10%)
HR-/HER2-	7 (17.5%)
Histologic grade	
Grade 1	6 (15%)
Grade 2	22 (55%)
Grade 3	12 (30%)
Ki-67	
Ki-67 <30	22 (55%)
Ki-67 ≥30	18 (45%)
Circulating NETs levels	
Low	23 (57.5%)
High	17 (42.5%)

Levels of circulating NETs were not statistically associated with age, stage, HR expression, HER2 expression, molecular subtype, histologic grade, and Ki-67. The associations between clinicopathological characteristics and levels of circulating NETs are summarized in **Table 2**.

**Table 2:** Associations between clinicopathological characteristics and circulating NETs levels*.

	Total number of patients	Low NETs		High NETs		*p*
		N	%	N	%	
Age						
<65 years	31	19	61.3	12	38.7	0.456
≥65 years	9	4	44.4	5	55.6	
Stage						
Early (stages I – IIA)	27	18	66.7	9	33.3	0.17
Locally advanced (IIB – IIIC)	13	5	38.5	8	61.5	
HR expression						
HR+	29	14	48.3	15	51.7	0.079
HR-	11	9	81.8	2	18.2	
HER2 expression						
HER2+	5	3	60	2	40	0.999
HER2-	35	20	57.1	15	42.9	
Molecular subtypes						
HR+/HER2-	28	14	50	14	50	0.191
HR+/HER2+	1	0	0	1	100	
HR-/HER2+	4	3	75	1	25	
HR-/HER2-	7	6	85.7	1	14.3	
Histologic grade						
Grade 1	6	1	16.7	5	83.3	0.100
Grade 2	22	15	68.2	7	31.8	
Grade 3	12	7	58.3	5	41.7	
Ki67						
Ki-67 <30%	22	12	54.5	10	45.5	0.755
Ki-67 ≥30%	18	11	61.1	7	38.9	

*Fisher’s exact test was used to evaluate associations between circulating levels of NETs and clinicopathological characteristics.

**Figure 1:**
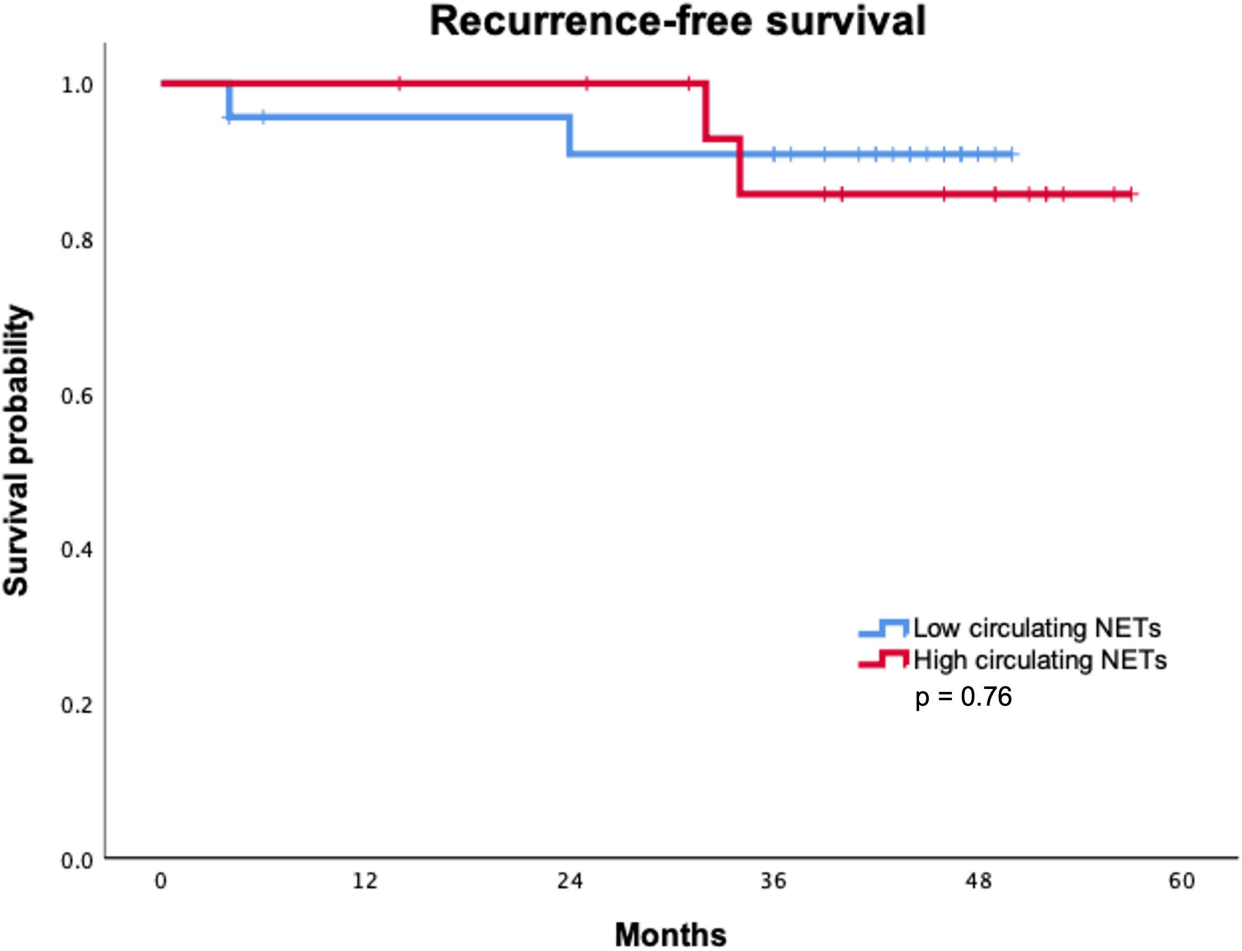
Recurrence-free survival according to levels of NETs.

With a median follow-up of 43.5 months, only two recurrences were identified in each group according to circulating NETs levels. Recurrence rate (p = 0.99) and site of recurrence (p = 0.99) were not statistically associated with circulating NETs levels (**Table 3**). Median RFS was not reached in either group. Overall, RFS was not statistically different between patients with high and low levels of circulating NETs. (**Figure 1**) RFS at 36 months was 90.9% for patients with low levels of NETs as compared to 85.7% in patients with high levels of NETs (p = 0.76). To date, no deaths have occurred in our cohort.

**Table 3:** Recurrence and circulating NETs levels*.

	Total number of patients	Low NETs		High NETs		*p*
		N	%	N	%	
Recurrence						
Yes	4	2	50	2	50	0.999
No	36	21	58.3	15	41.7	
Site of recurrence						
Locoregional	1	1	100	0	0	0.999
Distant	3	1	33.3	2	66.7	

*Fisher’s exact test was used to evaluate associations between circulating levels of NETs and recurrence outcomes.

## Discussion

In 2004, Brinkmann et al. first described NETs and their role in capturing and killing bacteria (9). The formation of NETs leads to a unique form of cell death characterized by the release of decondensed chromatin and granular contents to the extracellular space (10). Since the initial discovery of NETs, numerous reports have been published on the characteristics of NETs as well as on their protective role against pathogens and their pro-tumorigenic and/or anti-tumorigenic effects on cancer (5). Furthermore, patients with lung, pancreatic, and bladder cancer have been demonstrated to have higher levels of circulating NETs than healthy controls (11). Hence, the potential role of NETs as a diagnostic and/or prognostic biomarker is increasingly being studied in breast and other tumor types. In BC, inflammation-induced activation of neutrophils has been shown to awaken dormant BC cells by producing NETs, leading to lung metastases in mice models (12). Furthermore, *in vitro* studies using BC cell lines have shown that NETs upregulate the expression of pro-inflammatory factors associated with the epithelial-mesenchymal transition, an important mechanism promoting tumor cells’ metastatic potential leading to increased migratory and invasive abilities (13).

To the authors’ knowledge, this is the first prospective cohort to evaluate the association of circulating NETs at time of diagnosis with recurrence in patients with early BC. Our results suggest that circulating levels of NETs at time of diagnosis are not associated with recurrence or site of recurrence in women with early-stage BC. However, previous studies have demonstrated an association between high levels of NETs, recurrence, and site of metastatic lesions. Cai et al. showed that NETs content in breast tumor tissues was higher compared to the adjacent normal breast tissues. Furthermore, they reported higher recurrence rates in patients with higher NETs expression within the primary BC tumor, with a recurrence rate of 41.2% in the group with high NETs expression as compared to only 3.6% in the group with low NETs expression (14). Another prior study demonstrated that although NETs were scarce within primary BC tumors, they were avidly detected in several metastatic lesions, including those in the liver, lungs, bones, and brain. Interestingly, the concentration of NETs was higher in liver metastases than in other metastatic sites. Moreover, serum levels of NETs were significantly higher in patients with liver metastases as compared with those without metastases or those with metastases in other organs (15).

Contrary to prior reports in which more aggressive BC tumor subtypes were associated with higher levels of NETs, in our study, these characteristics were not associated with high levels of circulating NETs. In a previous study including 45 patients with BC who were followed up for five years, NETs in tumor tissue sections were significantly higher in younger patients and in those with triple-negative BC (14). Similarly, Park et al. found that number of NETs varied between tumors, with the highest numbers in triple-negative tumors (in primary BC tumors and lung BC metastases) and very low in luminal BC (16). A possible explanation for our contrasting results may be that primary BC tumors with aggressive clinicopathological characteristics may induce local NETs formation and consumption as an anti-tumorigenic mechanism to reduce tumor growth and dissemination, thus intratumoral NETs levels may be higher in more aggressive tumor types and lower in peripheral blood.

Prior studies have also demonstrated the controversial anti-tumorigenic role of NETs in melanoma, bladder, and head and neck cancer (17–19). Schedel and colleagues showed that the quantity of intratumoral NETs did not correlate with tumor progression of melanoma. Furthermore, *in vitro* data revealed that NETs attached to melanoma cells *via* integrin‐mediated adhesion leading to reduced melanoma cell migration and viability (17). Similarly, Li et al. demonstrated the cytotoxic role of NETs in the early stages of bladder cancer. In this study, NETs induced by Bacillus Calmette-Guerin stimulation exerted cytotoxic activity through enhanced apoptosis and cell-cycle arrest, and inhibited migration of bladder tumor cells *in vitro*. Additionally, *in vivo* NETs contributed to an increased immune response and direct tissue damage, thus preventing tumor growth (18). Finally, in the study by Millrud and colleagues, NETs were found to be produced intratumorally by activated neutrophils in head and neck squamous cell carcinoma (HNSCC). Activated neutrophils inhibited tumor cell migration, proliferation, and growth in HNSCC, suggesting a direct anti-tumor function of activated neutrophils mediated by NETs (19). While these findings demonstrate the anti-tumorigenic effects of NETs, the vast majority of evidence supports the pro-tumorigenic role of NETs.

Furthermore, the absence of prognostic association between circulating levels of NETs and recurrence in localized BC in our study may be partially due to the lack of a standardized definition of “high” levels of NETs, differences in detected components of NETs (DNA, NE, MPO, or citrullinated histones), and whether NETs should be measured intratumorally (primary tumor or metastatic lesions) or in plasma samples (7). Further research may help elucidate the best cut-off value for “high” NETs levels, if NETs should be measured intratumorally or in plasma, as well as defining the prognostic and/or predictive value of this biomarker. Moreover, the prospective validation and standardization of the assays quantifying NETs levels in human patients with BC will allow for the potential clinical implementation of this tool.

Other biomarkers are currently being investigated as potential surrogates of NETosis. It has been described that calprotectin (CP) and human neutrophil elastase (HNE) are secreted by the neutrophils during NETs formation resulting in HNE-derived CP neo-epitope protein fragments [CPa9-HNE] (20). The ex vivo findings of a study evaluating serum level of CPa9-HNE in patients with inflammatory bowel disease suggested that CPa9-HNE may be used as an adequate surrogate biomarker of NETosis (20). Furthermore, the biomarker potential of serum CPa9-HNE levels at baseline has also been evaluated in patients with metastatic melanoma treated with pembrolizumab. In this study, high pretreatment levels of CPa9-HNE were associated with worse progression-free and overall survival as compared to low levels of plasma CPa9-HNE (21). Therefore, diverse forms of detecting and quantifying NETs are currently being investigated, adding to the complexity of understanding and utilizing this potential biomarker in clinical practice. Furthermore, the prognostic value of other immune biomarkers such as the neutrophil-to-lymphocyte ratio (22, 23) and tumor-associated neutrophils (24, 25) are also currently being evaluated in BC.

Some limitations should be taken into account when interpreting these results: first, the small sample of patients included in this study with only four recurrences might limit the statistical power of our findings. Nonetheless, the recurrence rate in our small, mainly luminal cohort, resembles that of a larger retrospective study including 2,685 with a 10% recurrence rate and a higher incidence of recurrence among HER2+ and triple negative subgroup (26). Second, a median follow-up of 43.5 months may represent a short follow-up for BC patients who have a persisting and increasing risk of recurrence and death a long time after initial diagnosis, especially in the luminal subgroup of patients where recurrence continues to occur constantly from 5 to 20 years, even after 5 years of adjuvant endocrine therapy (27). Third, due to a small sample size, NETs levels were dichotomized, therefore, analyzing linear correlations of NETs, as a continuous variable, with clinicopathological characteristics and recurrence was not possible. However, the biggest strength of this study is its prospective nature. Furthermore, this is the first study reporting the lack of a statistically significant association between circulating levels of NETs and recurrence in patients with early BC, although it is important to highlight that only a limited number of recurrences have occurred within a short follow-up. Nonetheless, a longer follow-up may allow to detect differences in outcomes according to circulating levels of NETs. We consider important to report our results to encourage further, larger studies with the aim of potentially identifying the predictive and/or prognostic role of circulating NETs in patients with early BC.

## Conclusions

With a short follow-up and low number of events, our results suggest that circulating levels of NETs at diagnosis, measured as plasma NE-DNA complexes, are not associated with aggressive clinicopathological characteristics, recurrence, or site of recurrence in early BC. Our findings add to the heterogeneity of results available in the literature regarding the pro- and anti-tumorigenic roles of NETs in BC. Further research may help establish the prognostic and/or predictive value of this biomarker by defining a standardized cut-off value for NETs levels and site of NETs measurement (intratumorally or in plasma). The standardization of NETs levels quantification in human patients with BC will allow for the potential clinical implementation of this tool.

## Data availability statement

The raw data supporting the conclusions of this article will be made available by the authors, without undue reservation.

## Ethics statement

The studies involving human participants were reviewed and approved by the Institutional Review Boards (Ethics and Human Research Committees) at the Instituto Nacional de Ciencias Medicas y Nutricion Salvador Zubiran, Mexico City, Mexico, with the reference number 2114. The patients/participants provided their written informed consent to participate in this study.

## Author contributions

All authors listed have made a substantial, direct, and intellectual contribution to the work and approved it for publication.
